# Characterization of Simulated Liquid Radioactive Waste in a New Type of Cement Mixture

**DOI:** 10.1021/acsomega.2c05507

**Published:** 2022-10-04

**Authors:** Margit Fabian, Istvan Tolnai, Zoltan Kis, Veronika Szilagyi

**Affiliations:** Centre for Energy Research, Konkoly-Thege str. 29-33, Budapest1121, Hungary

## Abstract

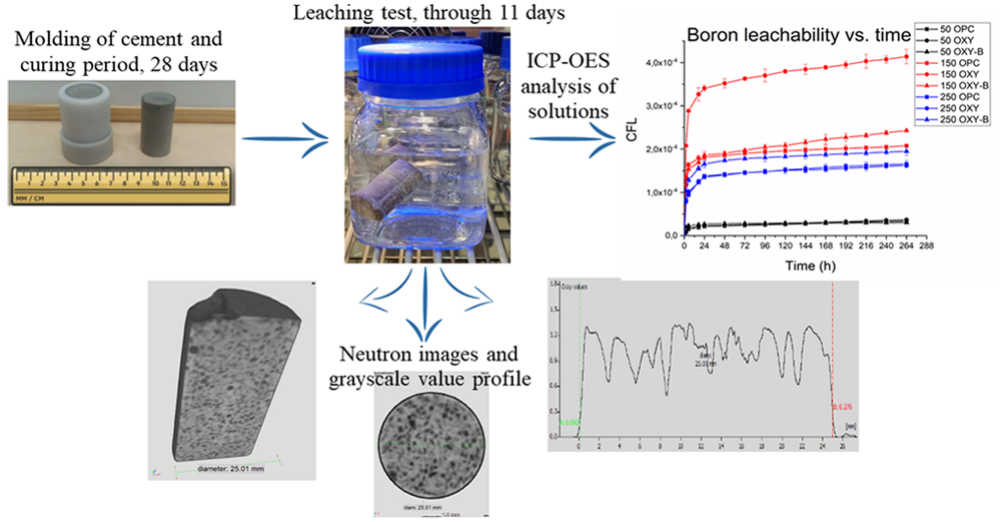

There is still a safety challenge for the long-term stabilization of nuclear waste. Due to its affordable price and easy manufacturing, cement is one of the most promising materials to immobilize a large volume of low- and intermediate-level radioactive liquid waste. To investigate the effect of borate on the cementation of radioactive evaporator concentrates and to provide more data for solidification formula optimization, simulated liquid waste in various concentrations was prepared. Different borate concentrations were solidified in ordinary Portland cement (OPC) and in two new cement compositions with water resistance and boron-binding additives. The chemical and mechanical properties were investigated for nine cementitious samples, together with three compositions in three concentrations. The leaching rate of the boron is lower in the case of a high strength cement mixture. The compressive strengths of the solidified waste correlate with the leaching rates of the boron. The leaching rates of Ca were changed with the cement composition and even with the boron concentrations; first, they were lower in the initial OPC in the case of the same boron concentration (50 g/L); second, they were lower at a higher boron concentration (250 g/L) for the OXY-B composition. The simulated liquid waste with higher boron concentrations solidified with newly developed cement composition (OXY-B) shows a homogeneous boron distribution in the volume of the cement cylinder both before and after leaching. The formulas of OXY and OXY-B developed in this application were effective for cementation of the simulated borate evaporator concentrates.

## 1 Introduction

Cements, due to their chemical, physical, and thermal stability, are widely accepted for immobilization of low- and intermediate-level liquid radioactive waste (LLW and ILW, respectively). Basic and applied research is conducted worldwide to study the solidification methods in an effort to find the best cement mixtures to understand their structure, determine solubility limits, and investigate chemical durability of host materials. During operation of nuclear power plants (NPPs) with WWER (water, water, energy, reactor—refers to Soviet design water-cooled, water-moderated, electricity-generating reactors), pressurized water reactors (PWRs) containing large amounts of liquid LLW/ILW containing inorganic boron compounds are produced and accumulated.^1^ Cement is useful for solidifying wastes such as boric acid and borate salts, but unfortunately, borates tend to inhibit or retard the hydration of the cement powder. Boric acid, which is the precursor of the borates, is used as a moderator in PWRs, and the borates which it forms are contained in both ion exchange resins and evaporation concentrates. The boric acid waste is generally solidified with a cement matrix, and this solidified radioactive waste is packed into containers that are isolated from the human environment by safe disposal.^2^ This study was carried out on the immobilization of evaporator concentrates containing borates in a new cement matrix and evaluation of its long-term stability.^3−5^

Borate waste concentrates vary in their content, ratios, and amount of salts according to conditions of operation, pretreatment, and coolant constituents (exp. ^137^Cs, ^60^Co). A simulated concentrated borate waste solution was used in the present study to evaluate the chemical characterization of the solidified borate-containing cement. Based on the literature, boric acid waste could contain ^90^Sr, ^137^Cs, ^51^Cr, ^60^Co, and other radionuclides.^4,6^

To ensure the radiological safety after waste disposal, this solidified radioactive waste is required to have some properties such as adequate mechanical strength, low leachability, beneficial effects during water immersion, and good durability. Our study takes into account the main guidance document of waste acceptance criteria (WAC)^7,8^ of the National Radioactive Waste Repository. Leaching is generally considered as the basic criterion to evaluate the safety, acceptability, and chemical behavior of the final waste forms in the disposal sites. A key property of any waste form is its leaching resistance, which determines how well the radionuclides of concern are retained within the waste form in a wet environment. Estimating the rate of leaching from a matrix during disposal is a key consideration in assessing an immobilization method. Low matrix solubility means reduced likelihood of radionuclide release. The neutron scattering provides information on the nature of the phase transitions and structure of condensed systems and gives information on the local environment and short-range structure. In this paper, we report neutron diffraction measurements of the bulk dried cements, with the aim to characterize the structure of cement compounds. Using neutron imaging, it is possible to assess the homogeneity of the boron content of the samples. Moreover, applying the prompt-gamma activation imaging (PGAI) method, localized elemental information can be gathered, which can support the findings from imaging tests.

This review aims at studying the solidification properties of the three different types of cement compositions and applying inactive “simulated” liquid borate in different concentrations. With a focus on the changes in mechanical and chemical properties, we would like to find the best association regarding the compositions and borate concentrations for a future method of stabilizing liquid radioactive waste.

## 2 Sample Preparation

### 2.1 Materials Used for the Experiments

This study was aimed to understand the chemical stability of borates in cementitious structures, as the most common hosting matrix for LLW/ILW. For the study, three types of cement mixtures were used. The basis of all three types was ordinary Portland cement and its variously doped versions. The basic compositions were a Portland 42.5 N cement (hereafter referred to as OPC). Additives (Sika Fume 10 wt %, Glenium 51 1 wt %, Sika DM 2 1 wt %)^9−11^ were added to OPC that changed the macro/micropore ratio of initial concrete from 70/30 to 3/97, increasing the chemical stability and ensuring water resistance, and the composition was named Oxydtron (hereafter referred to as OXY). In the Oxydtron composition, 2 wt % of the Sika Fume was replaced with Sika ViscoCrete to obtain the Oxydtron-B composition (hereafter referred to as OXY-B). The OXY-B has a double improved composition with explicit purposes to make the cement as watertight as possible to further reduce leachability and to prevent the release of bound boron from the system in aggressive conditions. The raw materials we used were dried powder mixtures, of which compositions were measured with X-ray diffraction analysis, and the results can be seen in Table 1.

**Table 1:** Phase Composition (in wt %) of Cements Used

	OPC	OXY	OXY-B
quartz (SiO_2_)	29	38	40
calcite (CaCO_3_)	15	17	10
dolomite (CaMg(CO_3_)_2_)	12	15	6
alite (Ca_3_SiO_5_)	39	25	39
brownmillerite (Ca_2_(Al,Fe)_2_O_5_)	2	2	3
gypsum (CaSO_4_·2H_2_O)	2	2	1
ettringite (Ca_6_Al_2_(SO_4_)_3_(OH)_12_·26H_2_O)	1		
Portlandite (Ca(OH)_2_)		1	1

### 2.2 Cementation Technology in Laboratory Conditions

Cements are used to immobilize both liquid and solid radioactive waste. Here, we focus on the solidification of liquid waste mixtures. Composition of the evaporator concentrates may vary, but the most significant components are NO_3_^–^ (5–72 g/dm^3^), H_3_BO_3_ (110–203 g/dm^3^), and NaOH/H_3_BO_3_ (0.95–1.21 molar ratio).^12^ In inactive model solutions, simulated liquid boric acid waste with 50, 150, and 250 g/L boric acid concentrations was prepared. During the experiments, the mixtures were designed to have a water/cement ratio (w/c) of 0.214, which is unusually low but not unprecedented.^13,14^ The addition of Glenium 51 allows for a high strength and low permeability final product even with such an extremely low water/cement ratio. Our nine prepared and studied samples are indicated in Table 2.

**Table 2:** Samples and Their Notations Used in This Study

	OPC	OXY	OXY-B
50 g/L H_3_BO_3_	50 OPC	50 OXY	50 OXY-B
150 g/L H_3_BO_3_	150 OPC	150 OXY	150 OXY-B
250 g/L H_3_BO_3_	250 OPC	250 OXY	250 OXY-B

This ratio differs from the traditional cement full hydration ratio (0.42) but showed good paste workability during the mixing and the mold-filling steps.^15^ The boron concentration covered a range of around 40 g/L, which is the average boron concentration of residues in NPPs’ evaporated sludge.^16,17^ Orthoboric acid powder (VWR Chemicals; CAS-No. 10043-35-3) was used and was mixed with demineralized water (DMW) (conductivity = 1.1 μS/cm, pH 7.5 at 23 °C). To increase the boric acid solubility in DMW, having completely homogeneous mixtures, and to overcome the cement-retarding effect of boric acid, granular sodium hydroxide (NaOH; VWR Chemicals; CAS-No. 1310-73-2) with a 1:1 NaOH/H_3_BO_3_ molar ratio was added to the solution.^18,19^ The cement powder was first poured into a mixer (HAUSER DM-601), and then the simulated liquid was added to the cement step by step. The mixtures were stirred mechanically (90 rpm for 12 min) at room temperature and humidity to obtain a completely homogeneous paste.^20^ The wet paste was filled into 2.5 cm diameter and 5 cm high polyethylene cylindrical molds.^21^ The molds were then shaken for 5 min to remove air bubbles from the paste. Then the molds were put in an incubator (VWR-INCU Line 68R) with a fixed temperature of 20 °C.^21^ The specimens were cured for 28 days, and then they were demolded by a manual hydraulic press (SPECAC 25T).^22^

## 3 Characterization of Solidified Cement Mixtures

### 3.1 Leaching Test

The measurement method for calculating the diffusion coefficient was performed according to the ASTM C1308-21 standard.^21^ According to this standard, the cylindrical solid samples with a 49.09 cm^2^ contact surface were immersed in 500 mL of DMW (leachant), and the resulting solutions (leachates) were changed at time intervals of 2, 5, 17, and 24 h and then daily for the next 10 days. After the leachate’s extraction, their pH values were measured with a calibrated electronic pH meter (XS pH 50+ DHS), where the changes of pH can be a sign of different phases being released into the leachates.^23,24^ All of the leachates were then filtered through a cellulose acetate membrane (FILTER-BIO; pore diameter of 0.45 μm) and acidified by 1 mol/mol % nitric acid (HNO_3_ 65%; Sigma-Aldrich; CAS-No. 7697-37-2). The treated samples were analyzed by an inductively coupled plasma optical emission spectrometer (ICP-OES; PerkinElmer Avio 200^25^).

### 3.2 Compressive Strength Tests of Hardened Cement

The compressive strength of hardened cement is one of the most important properties. After the curing time (28 days), 12 cylindrical specimens of three samples (50 OXY-B, 150 OXY-B, and 250 OXY-B) were tested and were designated “before leaching”. After the leaching test,^21^ 11 specimens of three samples (50 OXY-B, 150 OXY-B, and 250 OXY-B) were tested and were designated “after leaching”. The age of this cements was 39 days (28 days curing period and 11 days leaching period). We used a compressive strength testing machine, a calibrated press (Toni Technik Baustoffprüfsysteme GmbH), with geometry measuring tool, a calibrated digital caliper. The mass measuring device was a calibrated digital laboratory scale. Each device used meets the requirements of the European Standard EN 196-1:2016.^26^

### 3.3 Neutron Diffraction Measurements

Neutron diffraction (ND) experiments were performed at room temperature using monochromatic neutrons (λ_0_ = 1.069 Å) at the 2-axis PSD diffractometer of Budapest Neutron Centre in the momentum transfer range of *Q* = 0.45–9.8 Å^–1^.^27^ The cylinders—from OPC and OXY-B cements—with a 2.5 cm diameter were placed in the neutron beam and measured cc 24 h/each. Data were corrected for detector efficiency, background scattering, and absorption effects. The total structure factor, *S*(*Q*), was calculated with local software packages.

### 3.4 Neutron Imaging

**Figure 1:**
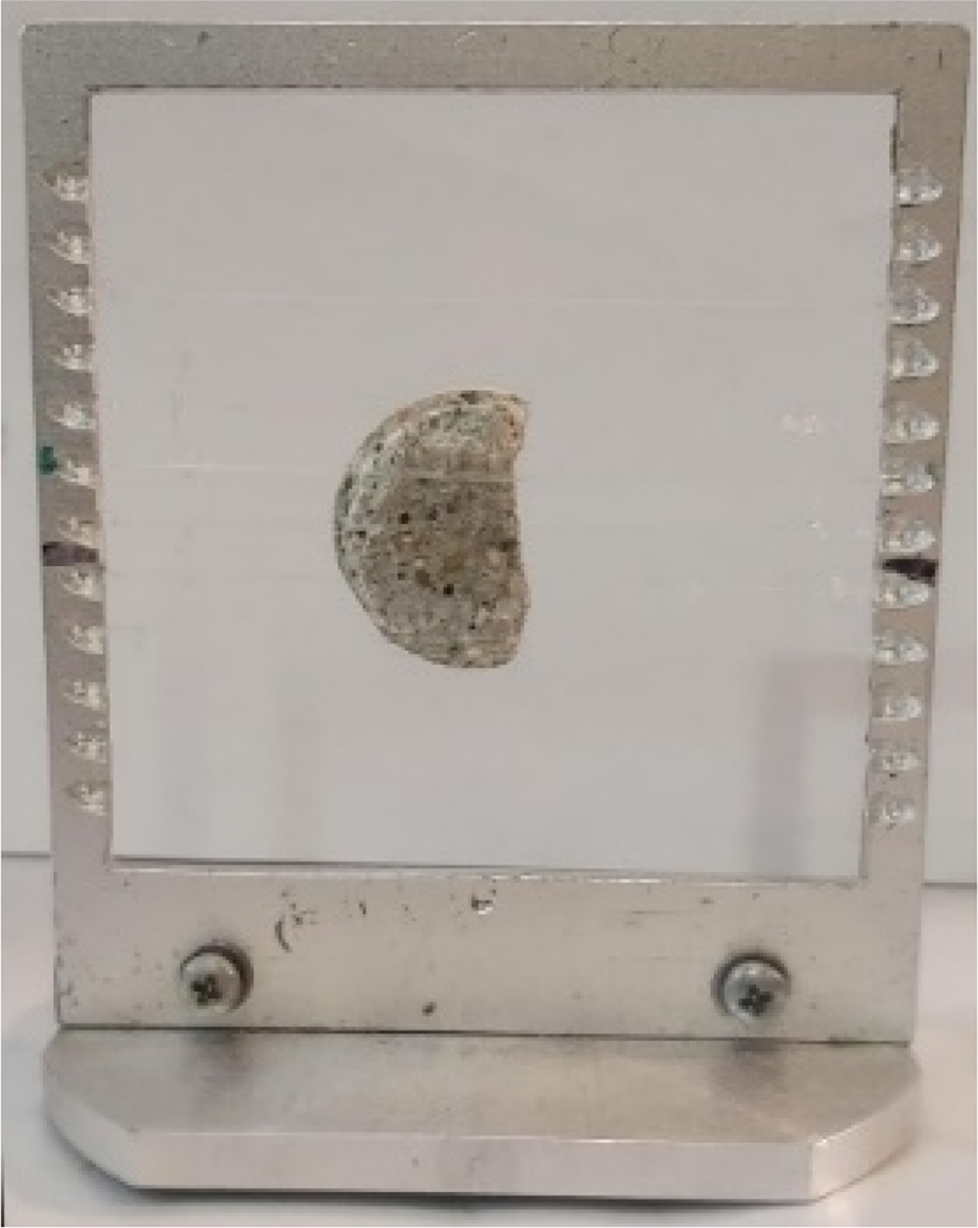
Cut from the cement cylinder (250 OXY-B) and its sample support used to gather the localized boron profile from PGAI measurements. The scan was accomplished along the vertical diameter of the sample. Photograph courtesy of co-author Zoltan Kis.

The neutron imaging measurements were carried out at the radiography/tomography (RAD) station of the Budapest Neutron Centre (BNC).^28^ Neutrons as electrically neutral particles usually easily penetrate a sample (see Figure 1), and the interactions that take place there cause the attenuation of the incoming neutron beam.^29^ The attenuated beam provides a shadow image on a detector screen, which can digitally be sampled by an optical system to match the attenuation values to the grayscale values (grv) pixelwise. Beam hardening (BH) means that lower energy neutrons in the beam tend to be absorbed more likely than higher energy ones, biasing so the reconstructed 3D tomographic slices have an imaging artifact called cupping.^30^ Since the linear attenuation coefficient of boron for thermal energy neutrons is rather high (102 1/cm), the BH effect is considerable for the white neutron beam of the RAD station. We used a beam hardening correction (BHC) method to provide unbiased 3D images. It was applied for the objects prepared from OXY-B with 250 g/L boric acid content so that the neutron attenuation values for several cement cylinders with known thicknesses were measured to calculate a calibration curve. This curve, which is valid only in the beam of the RAD station, was used in the 3D reconstruction process to provide beam-hardening-free images, which can then be used to assess the homogeneity of the boron content of the samples.

### 3.5 Prompt-Gamma Activation Imaging

The neutron elemental analysis measurements were carried out at the Neutron-Induced Prompt-Gamma-ray Spectroscopy-Neutron Optics and Radiography for Materials Analysis (NIPS-NORMA) station of the Budapest Neutron Centre (BNC).^31^ Prompt-gamma activation analysis (PGAA) is a neutron-based element analysis method^32^ for nondestructive quantification of major and minor components and several trace elements in the irradiated volume. PGAA relies on the radiative neutron capture process, which emits γ-rays with well-defined energies. The detected γ-rays can be assigned to the emitting elements, whereas from the intensities of these γ-peaks, quantitative analysis can be done. Prompt-gamma activation imaging is an extension of PGAA, where localized elemental information is gathered.^33^ By both limiting the impinging beam and positioning the sample in the beam, we can choose the probing areas of interest. Boron is an ideal element for PGAI because its neutron absorption cross section is exceptionally high (767 barn) compared to that for most of the other elements. In this case, we applied PGAI for an 8 mm high disk, which was cut out from the 250 OXY-B cement cylinder after the leaching process. We limited the beam size to 3 × 3 mm^2^ and scanned along the vertical diameter of the disk in eight successive steps (see Figure 1). In the resulting spectra, the boron to calcium ratio was calculated to be able to provide a localized profile of boron.

## 4 Results and Discussion

### 4.1 Consistency Test Results

The consistency of the fresh cement paste was measured according to the Suttard method.^34^ A 2.5 cm internal diameter and 5.0 cm high polyethylene cylinder was placed on a horizontal, smooth, and scaled base plate. The cylinder was filled to the top with fresh cement paste. Then the mold was lifted, and the cement paste flowed and spread on the base plate for 30 s, then we measured the maximum of the cement paste spread in the two directions, as presented in Table 3. The diameter of the spreading paste is calculated from the average of these two measurements.

**Table 3:** Consistency Measurements

sample	*x*+ (cm)	*x*– (cm)	*y*+ (cm)	*y*– (cm)
50 OPC	2.0	2.1	2.1	1.9
50 OXY	1.9	2.2	1.8	1.8
50 OXY-B	1.5	1.4	1.6	1.6
150 OPC	2.0	1.5	1.5	1.9
150 OXY	1.8	2.0	1.7	2.0
150 OXY-B	1.7	1.6	1.7	1.9
250 OPC	1.9	1.9	1.9	2.0
250 OXY	1.8	1.8	1.7	1.9
250 OXY-B	1.8	2.0	1.7	2.0

One of the WAC requirements is associated with the consistency of the cement pastes, which allows the free cavity volume to be filled inside the solidified waste in the containers. The Suttard consistency must be in the range of 1.5 to 2.0 cm. It can be seen from the Table 3 that the consistency parameters were not significantly affected by the cement compositions or borate concentrations, and even the numbers are in the range of WAC. The numbers show a random consistency without any trends.

### 4.2 Leaching Test Results

**Figure 2:**
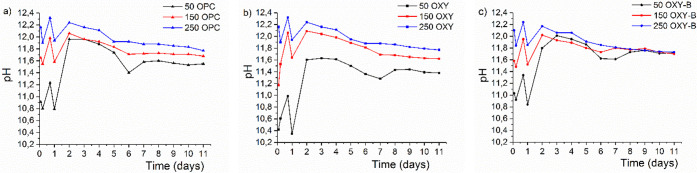
Time versus pH diagrams of the OPC (a), OXY (b), and OXY-B (c) leachates after sampling according to the ASTM C1308- 21 protocol.

The pH diagram for the three-cement series is presented in Figure 2. Based on the figures, changes for all three cements can be observed in the first period of the leaching. There is an active decreasing/increasing change up to day 2, and after that time, the pH shows a balanced downward trend. Comparing the three cement compositions (OPC, OXY, and OXY-B), the characteristics of pH are similar to each other.

Based on the standard procedure,^21^ the unitless incremental fraction of leached boron (IFL), which is the indexed parameter for sustainability comparison, during test interval *n* is calculated by
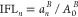
1where *a*_*n*_^*B*^ is the quantity of boron measured in the leachate from the *n*th test interval and *A*_0_^*B*^ is the quantity of boron in the solid specimen at the beginning of the test.

The cumulative fraction of leached boron until the *j*th interval (CFL_*j*_) is calculated by
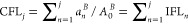
2Plotting the CFL values versus the cumulative time can provide a straightforward graphical comparison of leaching data from the various solidified cementitious samples.

**Figure 3:**
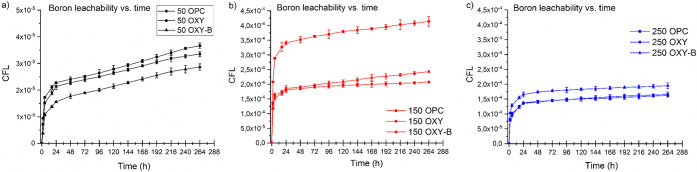
Cumulative leach fraction of B from the different cementitious waste form in different simulated liquid waste concentrations: (a) 50 g/L H_3_BO_3_, (b) 150 g/L H_3_BO_3_, and (c) 250 g/L H_3_BO_3_.

The results of leaching tests in DMW for OPC, OXY, and OXY-B cements are summarized in the panels of Figure 3. The panels show the cumulative leached fractions of B as a function of time for the three types of concentrations. The boron leachability is one of the most important parameters in the waste-form durability calculations. For all cement samples and for all of the boric acid concentrations, the boron dissolution shows dynamic activity between 0 and 24 h, followed by a slow rise, perhaps approaching a plateau. In the case of the 50 g/L simulated liquid waste (50 OPC, 50 OXY, and 50 OXY-B), the leached fraction of B shows similar rates for all three cement compositions (Figure 3a). Figure 3b,c indicates an increasing trend as the initial concentration of boron increases to 150 and 250 g/L in the simulated liquid waste. For all cement samples, the 150 g/L boric acid concentration shows the highest leach rate. A relatively higher jump was observed in the case of the 150 OXY sample, and below this, the leached fraction of B is in the same range for the OPC and OXY-B samples. Figure 3c shows the B leachability in the case of the 250 g/L simulated liquid waste, where a slight increase was obtained in the case of the OXY-B sample, but the leached fractions from OPC and OXY samples are similar. According to the B leachability results, there is almost no significant difference between cement specimens used for solidification of borates. In contrast, the different initial simulated liquid waste concentrations play a vital role in the leachability.

Based on the results obtained from the CFL calculations, it could be concluded that from the viewpoint of leached B, the most suitable cement composition is the OPC cement with a low 50 g/L simulated liquid waste concentration (Figure 3a).

**Figure 4:**
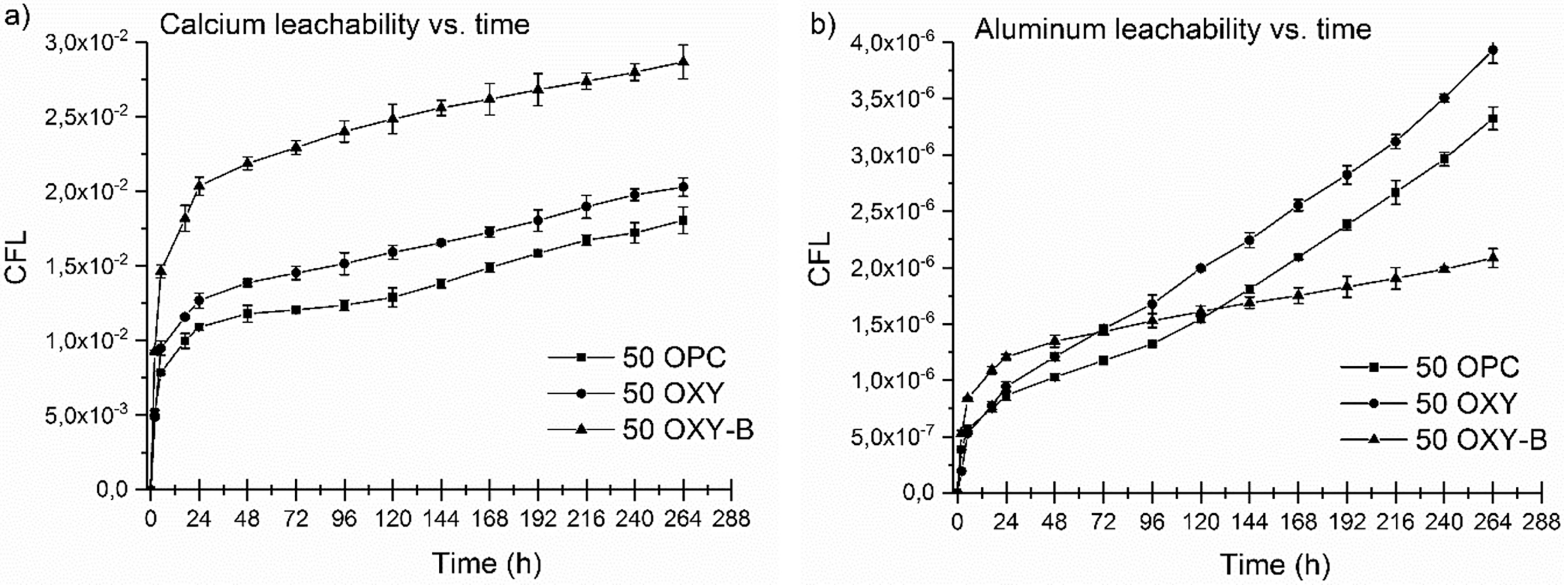
Cumulative leach fractions of Ca (a) and Al (b) from the different cementitious waste forms with the same simulated liquid waste (50 g/L H_3_BO_3_) concentrations.

The cumulative leaching fraction values of the Ca and Al elements within the leached samples, as obtained from ICP-OES, are presented in Figure 4. Ca is the major cement component; Al is the secondary element in the cement mixture. In Figure 4, we present the Ca- and Al-leached fractions in the same simulated liquid waste concentration (50 g/L H_3_BO_3_) with different compositions and in the function of time. Calcium should show the highest elemental leach rank in cementitious waste form.^35^ A significant increase in leached fraction of Ca was obtained for the 50 OXY-B samples (Figure 4a), while for the 50 OXY and 50 OPC samples, the leached fraction was closer to each other. The cumulative leached fraction obtained for Al (Figure 4b) was much lower than that for Ca, and the trends between the compositions were similar; even in the case of 50 OPC and 50 OXY samples at the last part of the leaching experiments, the dissolution was much more intense than that in the case of the 50 OXY-B sample.

**Figure 5:**
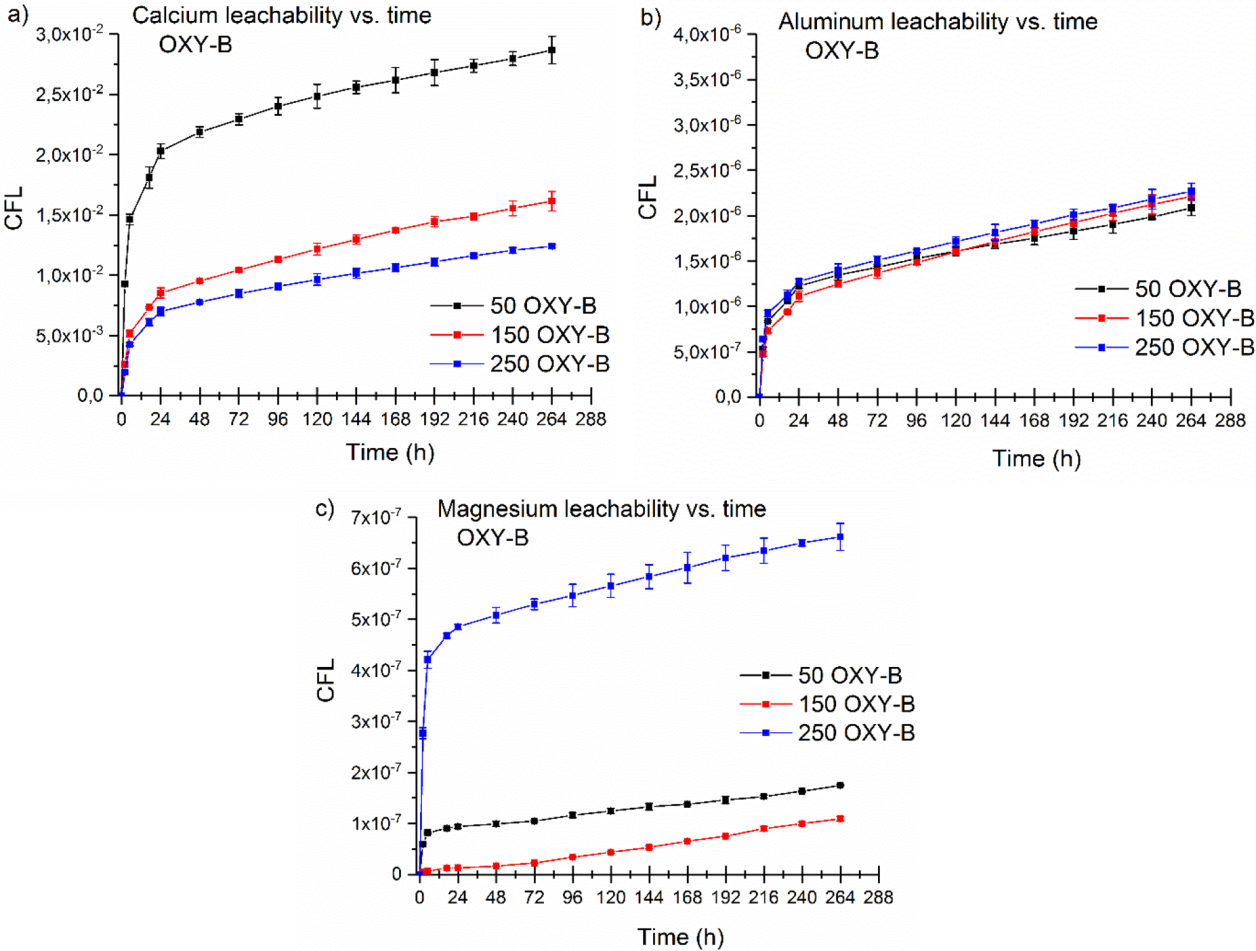
Cumulative leach fractions of Ca (a), Al (b), and Mg (c) from the OXY-B cement composition with different initial concentrations (50 g/L H_3_BO_3_ (black), 150 g/L H_3_BO_3_ (red), 250 g/L H_3_BO_3_ (blue)).

The cumulative leached fraction values for Ca, Al, and Mg were compared for different simulated liquid waste concentrations but with the same cement composition, OXY-B (Figure 5). The leached fraction of Ca shows a decrease with increasing of H_3_BO_3_ concentrations; 50 g/L > 150 g/L > 250 g/L (Figure 5a). This high leaching expectation of calcium is seen in the result of the leaching test shown in Figures 4a and 5a. However, the comparison results of the CFL for calcium leachability for OXY-B composition (Figure 5a) show that the cementitious specimens containing a lower initial H_3_BO_3_ concentration (50 OXY-B) have a calcium leaching 2–3 times higher than that of the higher initial H_3_BO_3_ concentration samples (150 OXY-B and 250 OXY-B). The general higher calcium leaching is due to retarding effects of boron on the cement hydration and the effect of boron leaching on the porosity of the cementitious matrix.^36,37^

The release of Al was not influenced by the concentrations, and the cumulative leached fractions were almost similar (Figure 5b). Based on the results of this study, aluminum shows low leachability, which is in agreement with the previous similar studies.^35^ However, the low amount of aluminum release can mostly take place to compensate the non-equilibrium charge induced by boron replacement in the cementitious matrix.^38^ In all of the studied cementitious specimens, magnesium shows the lowest leachability (Figures 5c). Low leachability was observed for Mg, but a slightly higher leached fraction for the 250 OXY-B sample was measured.

Leachability of the Ca is a key factor, due to the main composition of the cements. Low cumulative leaching fractions were found for the OPC cement in the case of the same initial H_3_BO_3_ concentration (50 OPC, Figure 4a) and for highest initial H_3_BO_3_ concentration in the case of the same cement composition (250 OXY-B, Figure 5a).

### 4.3 Compressive Strength Test Results

**Figure 6:**
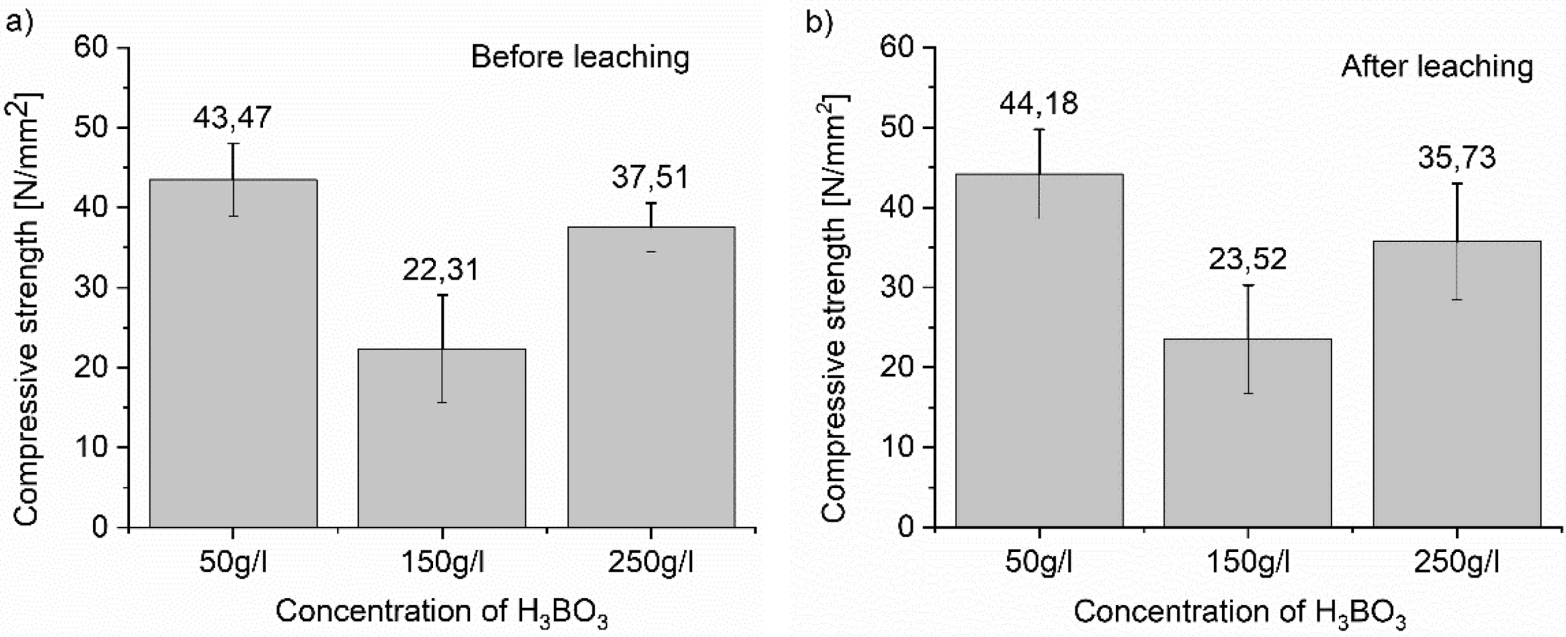
Compressive strength data for OXY-B cement, before (a) and after (b) the leaching experiments.

Most of the additives affect the compressive strength; therefore, the most improved composition, OXY-B samples, was investigated from this viewpoint. The compressive strength measurements were obtained for the OXY-B series before and after the leaching test, after 28 days and after 39 days, respectively. Increasing boric acid content in OXY-B cement waste forms decreased the compressive strength values (Figure 6), according to the order 50 OXY-B > 250 OXY-B > 150 OXY-B. There are quite large differences between the concentrations, but the parallel results of the same sample before and after the leaching are very similar. It means that the leaching conditions do not significantly affect the strength of the samples, which in terms of application, is a significant result. After the leaching test, the strengths of the 250 OXY-B samples decrease 5%, and the strengths of the 50 OXY-B and 150 OXY-B samples increase 1.5 and 5%, respectively.

Several studies discussed that, with reaction of the boric acid and calcium hydroxide (produced during the cementations), calcium borate were produced, which is insoluble; it acts as a barrier and retards the diffusion of elements.^39^ In the case of B, the lower leached fraction was associated with the highest compressive strengths in the case of 50 OXY-B. However, the leachability of B correlates with the obtained mechanical data. The WAC requirement for the compressive strength is minimally 10 N/mm^2^, as each sample surpasses the expected value.

### 4.4 Neutron Structure Factors of the Cements

**Figure 7:**
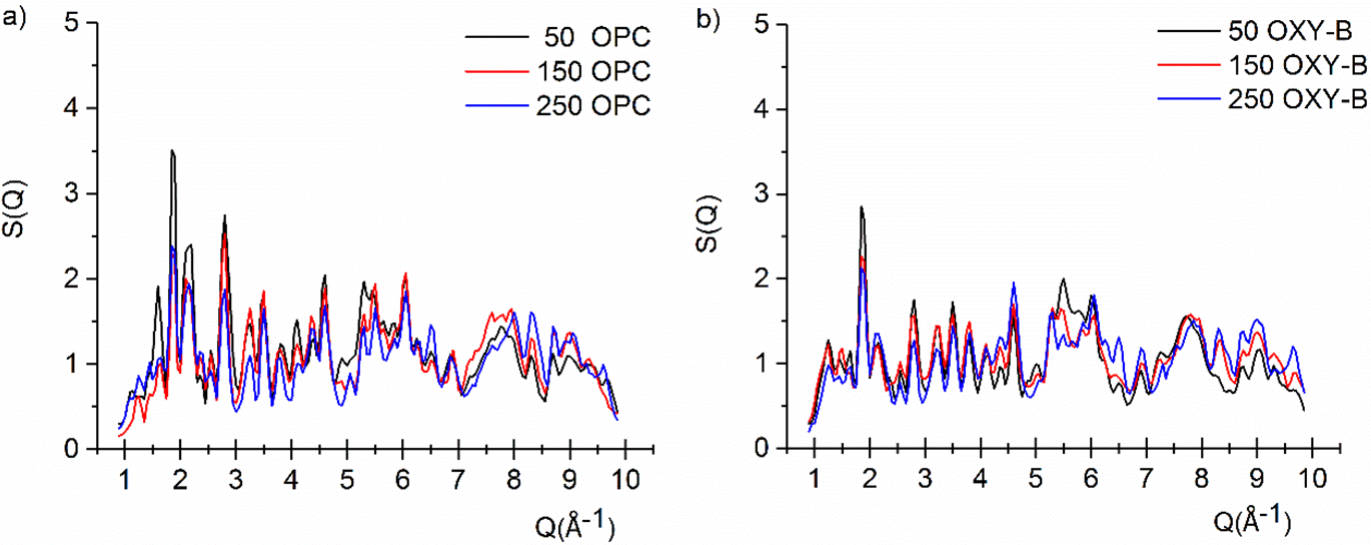
Total structure factors for OPC (a) and OXY-B (b) samples for all three boric acid concentrations.

The cement samples at the end of the curing period (28 days) were put in the oven at 100 °C for 2 days. The OPC and OXY-B cement samples used in neutron diffraction measurements were made from cement powder mixed with H_2_O (DMW). The incoherent scattering from the hydrogen in the samples prepared with H_2_O covered up the pattern from coherent scattering in the spectra, and only approximate results could be obtained on these samples. Figure 7 shows the neutron structure factors^40^ of the OPC and OXY-B samples.

Differences can be observed in the low *Q* range between the two series, but within a series, the characteristics of the spectra are similar. From neutron diffraction, we obtained mixed phases; amorphous phases can be identified as indicated by the broad distributions, and crystalline phases are also present. The broad amorphous component is attributed to the calcium–silicate–hydrate (C–S–H) gel^41^ with a typical Ca/Si molar ratio of 1.7 ± 0.1^42^ and may also contain a contribution due to strongly bound water on the gel surface. The sharp peaks could be attributed to the unreacted crystallites of the cement components. Due to the resolution limitation of our instrument, we do not have the possibility to separate these various components of the scattering. Several diffraction peaks are present in both samples’ series, varying only in intensities. In the case of the OPC series, some of the peak intensities have been enhanced due to the contribution of H_2_O. However, the peaks are present with almost the same intensity, indicating that the basic structure of the crystalline component has not changed significantly.

### 4.5 Neutron Imaging Results

**Figure 8:**
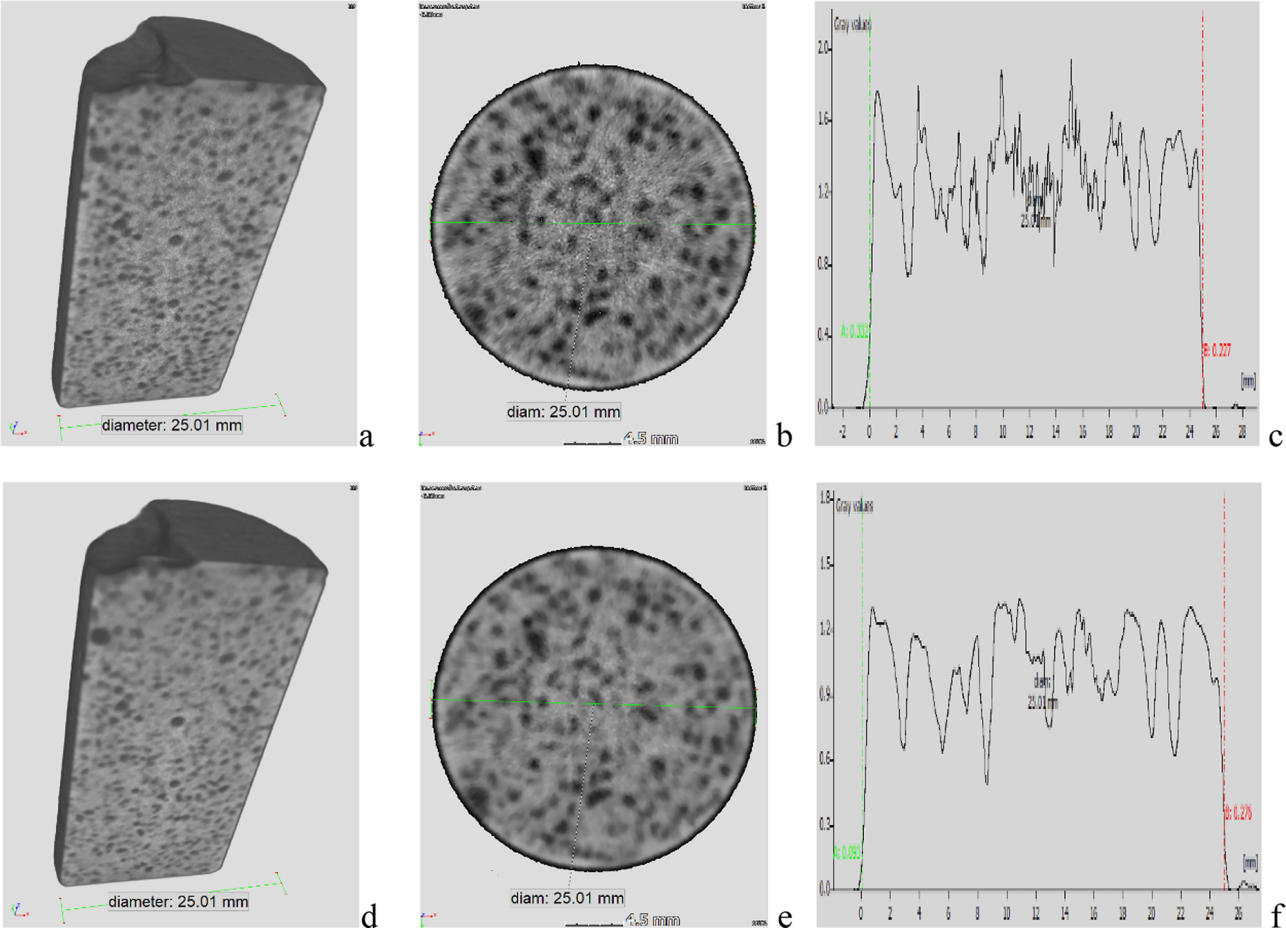
Neutron tomography results for spatial distribution of boron in a 250 OXY-B cylinder (a) 3D cut before leaching, (b) *xy* cut before leaching, (c) grayscale value profile along the diameter in the *xy* cut before leaching, (d) 3D cut after leaching, (e) *xy* cut after leaching, and (f) grayscale value profile along the diameter in the *xy* cut after leaching.

We provide boron homogeneity results showing beam hardening corrected 3D neutron tomographic images for a cement cylinder prepared from OXY-B with a 250 g/L H_3_BO_3_ solution. The same BHC calibration curve was used for the sample before and after leaching. One can see the dark spots in Figure 8, where there are grains/particles or pores in the volume, while the brighter areas show where the boron is located. Based on the 3D cuts and the plot profiles, we can state that the spatial distribution of the boron is rather homogeneous in the volume of the cement cylinder both before and after leaching. It strengthens the idea of the very low mobility of boron in this matrix.

### 4.6 Prompt-Gamma Activation Imaging

**Figure 9:**
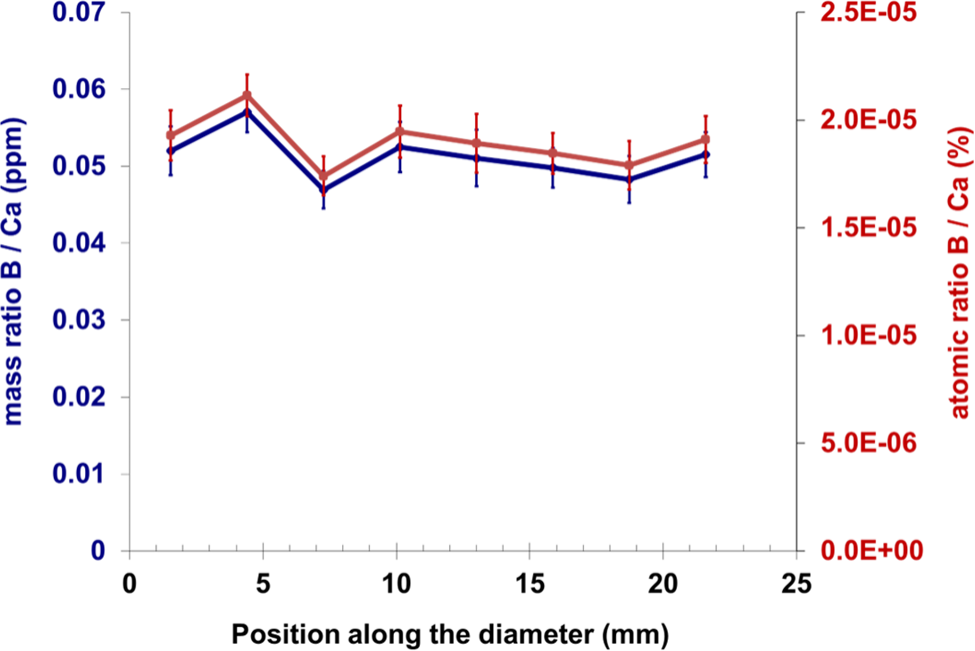
B/Ca atomic and mass ratio profiles from a PGAI scan along the diameter of a disk in eight successive steps. The disk was cut out from the cement cylinder (250 OXY-B) after the leaching process, and the beam size was limited to 3 × 3 mm^2^. Error bars are for 2σ confidence interval.

The results from the PGAI scan supported the findings from the neutron tomography. In Figure 9, the B/Ca atomic and mass ratios show a rather constant profile along the diameter of the cylinder. The slight changes could be attributed to the non-homogeneous structure of the matrix because boron is not absorbed by particles/grains, which can bias the ratio between B and Ca.

## 5 Conclusions

In this study, we focused on the investigations of nine inactive cementitious samples to find the best cement composition which can solidify as much borate as possible from simulated liquid waste. Based on Portland 42.5 cement, two cement compositions were developed for this application. The technological compliance was assessed by compressive strength tests and consistency measurements. We proved that all of our samples meet the consistency requirements. Considering different borate concentrations within the same OXY-B composition, each cement specimen meets the strength criteria.

The chemical characterization of solidified mixtures relied on leaching data and neutron-based investigations. The leached fraction of boron showed an increasing trend in all cements up to 24 h; after that, a barely noticeable growth was detected. The highest cumulative leached fraction for boron was obtained for the 150 g/L initial concentration and the lowest for the 50 g/L H_3_BO_3_ initial concentration. No significant differences were observed between the cement compositions, and only one significant change was measured in the case of 150 OPC. Both the neutron imaging and elemental analysis results showed a homogeneous boron distribution in the 250 OXY-B sample, which is the most extreme combination due to the high H_3_BO_3_ concentration associated with a newly developed OXY-B cement composition (presumably, there is a homogeneous boron distribution in the other samples with lower concentrations, too). The homogeneous boron distribution was not affected by the 11 days of leaching. The presence of the calcium–silicate–hydrate gel was proven by neutron diffraction measurements. We worked with inactive, simulated liquid waste; therefore, the chemical requirements of the WAC were not fulfilled.
